# Supplementary material for: Splice-Junction-Based Mapping of Alternative Isoforms in the Human Proteome
Source: Cell Rep. Author manuscript; Available in PMC 2020 Jan 15. (PMC6961840; doi:10.1016/j.celrep.2019.11.026)
Supplement: 2 [file NIHMS1546469-supplement-2.zip › DF1/PXD010154/Thyroid/Thyroid_41_RPS15_FIPLRSRAEEEADLPQVHLPR.pdf]

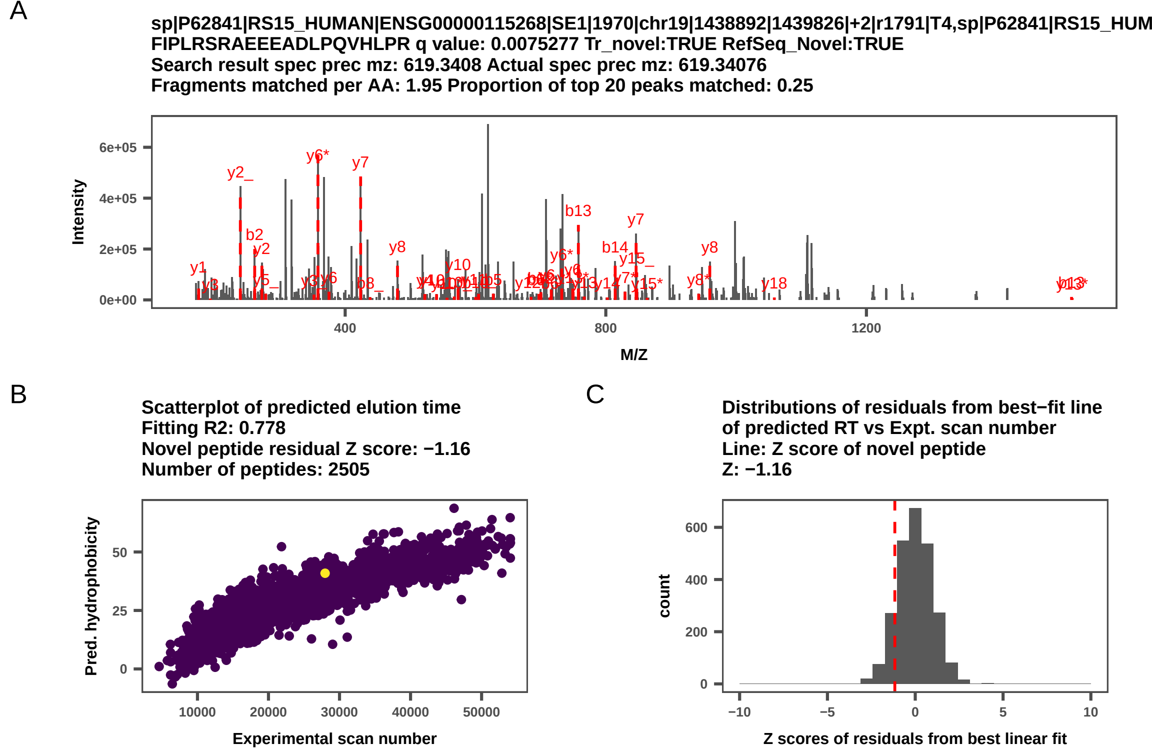
A
sp|P62841|RS15_HUMAN|ENSG00000115268|SE1|1970|chr19|1438892|1439826|+2|r1791|T4,sp|P62841|RS15_HUM
FIPLRSRAEEEADLPQVHLPR q value: 0.0075277 Tr_novel:TRUE RefSeq_Novel:TRUE
Search result spec prec mz: 619.3408 Actual spec prec mz: 619.34076
Fragments matched per AA: 1.95 Proportion of top 20 peaks matched: 0.25
Intensity
6e+05
4e+05
2e+05
0e+00
y1
y2_
b2
y2
y6*
y7
y8
y10
b13
y7
b14
y15_
y8
y18
400
800
1200
M/Z
B
Scatterplot of predicted elution time
Fitting R2: 0.778
Novel peptide residual Z score: −1.16
Number of peptides: 2505
Pred. hydrophobicity
50
25
0
10000
20000
30000
40000
50000
Experimental scan number
C
Distributions of residuals from best−fit line
of predicted RT vs Expt. scan number
Line: Z score of novel peptide
Z: −1.16
count
600
400
200
0
−10
−5
0
5
10
Z scores of residuals from best linear fit
